# Telephone health services in the field of rare diseases: a qualitative interview study examining the needs of patients, relatives, and health care professionals in Germany

**DOI:** 10.1186/s12913-018-2872-9

**Published:** 2018-02-09

**Authors:** Ana Babac, Martin Frank, Frédéric Pauer, Svenja Litzkendorf, Daniel Rosenfeldt, Verena Lührs, Lisa Biehl, Tobias Hartz, Holger Storf, Franziska Schauer, Thomas O. F. Wagner, J-Matthias Graf von der Schulenburg

**Affiliations:** 1 CHERH - Centre for Health Economics Research Hannover, Leibniz University Hannover, Otto-Brenner-Straße 1, 30159 Hannover, Germany; 2 ZQ - Centre for Quality and Management in Healthcare, Medical Association of Lower Saxony, Berliner Allee 20, 30175 Hannover, Germany; 3 ACHSE – Alliance for Chronic Rare Diseases, DRK-Clinics Berlin, Drontheimer Straße 39, 13359 Berlin, Germany; 4 IMBEI - Institute for Medical Biometry, Epidemiology and Informatics, Obere Zahlbacher Str. 69, 55131 Mainz, Germany; 5 Department of Dermatology, Freiburg Center for Rare Diseases, University Medical Center, University of Freiburg, Hauptstraße 7, 79104 Freiburg, Germany; 6 University Centre for Thorax Oncology, University Clinic of the Johann Wolfgang-Goethe University, Theodor-Stern-Kai 7, 60559 Frankfurt am Main, Germany

**Keywords:** Rare diseases, Telemedicine, Health-seeking behaviour, Helpline, Health information

## Abstract

**Background:**

Rare diseases are, by definition, very serious and chronic diseases with a high negative impact on quality of life. Approximately 350 million people worldwide live with rare diseases. The resulting high disease burden triggers health information search, but helpful, high-quality, and up-to-date information is often hard to find. Therefore, the improvement of health information provision has been integrated in many national plans for rare diseases, discussing the telephone as one access option. In this context, this study examines the need for a telephone service offering information for people affected by rare diseases, their relatives, and physicians.

**Methods:**

In total, 107 individuals participated in a qualitative interview study conducted in Germany. Sixty-eight individuals suffering from a rare disease or related to somebody with rare diseases and 39 health care professionals took part. Individual interviews were conducted using a standardized semi-structured questionnaire. Interviews were analysed using the qualitative content analysis, triangulating patients, relatives, and health care professionals. The fulfilment of qualitative data processing standards has been controlled for.

**Results:**

Out of 68 patients and relatives and 39 physicians, 52 and 18, respectively, advocated for the establishment of a rare diseases telephone service. Interviewees expected a helpline to include expert staffing, personal contact, good availability, low technical barriers, medical and psychosocial topics of counselling, guidance in reducing information chaos, and referrals. Health care professionals highlighted the importance of medical topics of counselling—in particular, differential diagnostics—and referrals.

**Conclusions:**

Therefore, the need for a national rare diseases helpline was confirmed in this study. Due to limited financial resources, existing offers should be adapted in a stepwise procedure in accordance with the identified attributes.

## Background

Rare diseases (RDs) are predominantly very serious and chronic diseases as approximately 80% are genetic in origin. Therefore, they often have a negative impact on the life expectancy and quality of life of those affected. In particular, people suffering from very rare RDs occurring once among 100,000 people are in danger; 5000 to 8000 different RDs have been detected thus far, accounting for 6% to 8% of the population [1]. Therefore, approximately 350 million people worldwide suffer from an RD, and half of them are children. People affected often struggle to obtain a proper diagnosis as healthcare providers have little experience of these conditions, and there is limited research evidence available. In addition, treatments, which, when available, are very expensive. These hurdles trigger an odyssey through health service systems and, in this context, the search for helpful health information. However, useful, high-quality, and up-to-date information is often hard to find [2].

The following article examines the potential of telephone services in satisfying this desire and elicits the revealed health-seeking behaviour. It introduces ‘helplines’ as services solely offering telephone-based information. Different types of information are differentiated, such as references, counselling, and/or medical information. Comparing different information access points, helplines are currently often used after the Internet and booklets [3]. Per the findings of Mevissen et al. [4]. Internet information can be delivered in addition to telephone information but should not be seen as an adequate replacement. Highlighting the importance of helplines aligned with other information access points, Ekberg et al. [5] offer an explanation for these findings as they show that emotional support needs are often intertwined with information- or advice-seeking needs.

### The case of helplines in the literature

To present helpline research, a brief indicative literature review was conducted by searching the MEDLINE database. The DIMDI (Deutsches Institut für Medizinische Dokumentation und Information, German Institute of Medical Documentation and Information) platform was used as a search tool. In all, 233 results were generated, including the keywords ‘helpline’ and ‘help line’ (search date: 27 May 2016). Results concerning animal research were excluded. Exclusion and inclusion criteria were set as displayed in Table 1.

**Table 1:** Inclusion and exclusion criteria

	Inclusion criteria	Exclusion criteria
Population	All potential patients (not only focusing on rare diseases), family members and physicians	Studies regarding animals
Intervention	Telephone services	Email services, Internet platforms
Outcome	Examination or improvement of helpline service or design Evaluation of caller behaviour	Examination of helpline callers to examine their general health behaviour (not offering additional knowledge to helpline design)
Publication type	Caller statistics, interviews and reviews	Interventional studies
Language	English, German, and French	All other languages
Time frame	All publications up to May 27th, 2016	None

Telephone services were often mentioned as being useful as a recruitment tool for participants of other health-related studies or the evaluation of health policies. These studies were excluded as they are not relevant in this context, leaving a total of 83 results. Findings are often based on the evaluation of caller statistics illuminating the profile of callers (65%, 54/83). Besides, many studies conducted a thematic analysis of telephone conversations. Questions were raised about caller satisfaction, perceived effectiveness, and support provision. Only five studies used interviews as a research method. Another five studies conducted structured literature reviews. Health professionals were rarely included. Most of the studies evaluated helplines addressing issues such as tobacco cessation (15%) [6–19], psychological problems (13%) [20–31], cancer (14%) [32–43], and family planning and sexuality (13%) [44–54]. Trials dealing with the specific concerns of RD patients and their physicians could not be determined. Building an argument for telephone services in general proves to be very difficult as helplines contribute to very heterogeneous health-related outcomes. Two studies, for example, use a successful referral to an appointment as an endpoint for the measurement of effectiveness of a helpline on sexuality and family planning [55, 56]. Tobacco cessation helplines with proactive counselling monitor the chance of quitting, [57] and psychological helplines the number of suicide preventions [58]. Other benefits are rather intangible and therefore difficult to measure, confronting helpline research with the criticism that little robust evidence is generated [59]. For example, general practitioner (GP) helplines offer access to the health care system after closing hours [60]. Furthermore, users of helplines for family planning, addiction, and violence perceive a telephone service as beneficial due to the ability to talk anonymously about delicate health issues [54, 61]. In this regard, helplines offer the chance to identify as well as bridge gaps between patients and health care service systems and, thus, play an important role in health care systems. Therefore, existing research suggests potentials of RD helplines; however, this hypothesis still needs verification. Ferreira et al. [62] report that helplines designed after patients’ needs contribute to the overall satisfaction of citizens with health care systems and their effectiveness, therefore highlighting the need for further research on what exactly is needed. The presented literature suggests that there are differences in themes, staffing and structuring of helplines, which should be thoroughly thought of. Encouraged by these findings, we further investigated potentials of RD helplines, resulting from the gap between information offering and need, to further improve health-seeking processes of people affected by RD.

As the literature search did not reveal any RD specific publications, we added a targeted manual search aiming for literature on RD helplines. Only one paper from Houÿez et al. [63], summarizing caller statistics of the European Network of Rare Disease Help Lines (ENRDHL), was found. However, the retrospective design did not allow for any recommendations improving existing structures. Additionally, two oral presentations [64, 65] mentioning RD helplines as part of national information provision on RD were listed. Other initially identified literature reported on non-specific disease helplines. Besides, Iskrov and Houÿez [66] also analyzed ENRDHL callers. On the other hand, Mazzucato et al. [67] stressed the need for telephone services parallel to other information systems. They forward an argument for bundling RD helpline services at a national level, noting that feedback concerning the functioning of RD policies can be retrieved immediately. However, data on the operational realization has not been raised.

### Political endorsement

Political processes have initiated national efforts targeted towards the improvement of the overall situation of individuals suffering from RD. In the EU, for example, policy proposals for the improvement of the overall situation were summarized in the European Commission Communication on RD in November of 2008 [68] and the European Recommendations to Member States by the Council of Ministers in June of 2009 [1]. Consequently, EU member states were encouraged to develop national plans to enact these requirements. Germany, for instance, published a National Plan for RD, the National Action League for People with RD (Nationales Aktionsbündnis für Menschen mit Seltenen Erkrankungen, NAMSE), in August of 2013 including 52 policy proposals [69]. Part of this action plan is the improvement of knowledge transfer through the expansion of disease-spanning, quality-orientated, and Internet-based information databases and systems. Towards this goal, the Central Information Portal for RD (ZIPSE – www.portal-se.de) was implemented. This is in line with an increasing international effort targeted towards the improvement of information structures. A growing number of national and transnational RD Internet platforms evolved [70].

Alongside an Internet-based information provider, the implementation of a telephone-based information service has been conceived as an alternative information access point. The Commission Communication also mentions the need for national RD helplines. To this end, the ENRDHL was named and created in the context of the European Rare Disease Solidarity Project (RAPSODY, September of 2006 to April of 2008). The focus of this initiative is the improvement of quality of services and providing a unified standard by sharing the experiences of European RD telephone helplines [71].

This demand is clearly highlighting the crucial point. Helplines do already exist, as an Orphanet list on international RD helplines [70], however, projects such as RAPSODY show that there are efforts necessary to set common standards. Besides, ENRDHL consists solely of members from eight countries, plus two countries in which helplines are still under construction [63]. Germany is not yet listed. However, NAMSE policy proposals 38 and 39 include the analysis of the implementation of a telephone service. NAMSE recommends to set up “[…] a pilot project to determine which target groups would make best use of such a hotline, what types of questions would most often be posed and what answers can best be delivered to these questions. This information would serve to determine the probable frequency and type of questions and how to plan to best meet these demands.” [69].

### The present article

The literature search shows how important telephone services are for health care service provision. Besides, there is little knowledge on RD helplines. Particularly, the perspective of potential callers has not been chosen in helpline research so far. This enables us to capture all relevant aspects for the design of a satisfying and effective RD helpline. The secondary aims of the underlying article were to add to the existing literature and to allow for substantiated decision-making in the political context aiming for the improvement of information provision for patients, family members and physicians. In this regard, the major aim of the study was to examine the needs of patients, relatives and health care professionals for a telephone based health service for RD in Germany triangulating perspectives of all potential callers, interviewing individuals suffering from RDs, their relatives, as well as health care professionals (HCPs).

## Methods

### Setting

The interview study was conducted as part of ZIPSE project, aiming for the implementation of an Internet platform for information on RD and considering a telephone service as an additional information access point. Patient and relative interviews were carried out between March and November of 2014 by three interviewers. HCP interviews were carried out by two interviewers between April and October of 2014. A qualitative setting was chosen as this design not only offers the opportunity to provide a first impression of a possible need structure but also drafts an RD helpline through the eyes of those interviewed. Forty Interviews with patients and relatives were conducted face-to-face and telephone-based in 29 cases. One interview could not be evaluated as the record was not readable due to technical difficulties, leaving 68 recorded interviews from patients and family members. In the case of physicians’ interviews, 39 interviews were conducted. Only seven interviews were carried out using the telephone to avoid long travel and scheduling on short notice. A change of interview medium was necessary due to the broad geographic coverage of the study within Germany.

### Participants

Patients and relatives were recruited through the Freiburg Centre for RD located at the Department of Dermatology of the University Medical Centre at the University of Freiburg and through RD self-help groups. The equal coverage of the many disease groups summarized under the broad definition of RD was targeted. Therefore, six participants were equally chosen among genetic skin disorders, skeletal dysplasias, neuromuscular disorders, genetic eye disorders, disorders of the connective tissue, genetic kidney diseases, cystic fibrosis and lung diseases, inherent disturbance of haematopoiesis, immunodeficiencies, inherent metabolic disturbances, and genetic diseases of the digestive tract. However, interview results showed in nearly all cases a complex, polysystemic pattern of involvement. At least nine patients had experienced a long process of diagnosis with duration of search for a diagnosis of more than 10 years. Thus, adding 66 patient and relative interviews to 10 interviews with prolonged diagnosis, a total sample of 76 patients was planned to be recruited. Nevertheless, interim analysis showed that upon saturation of interview data, a smaller sample would suffice. Further interviewing was not performed as this would not have led to expanded knowledge on the research subject. The final sample contains 55 individuals living with an RD and 13 family members.

For HCP interviews, five different groups were incorporated: GPs, specialists, physicians working in a hospital and medical therapeutic practitioners (MTP). In this context, the term “clinicians” refers to those physicians working in a clinical surrounding. In Germany this subgroup needs to be distinguished from “specialists” who have settled in a private practice. RD guides differed in qualification (e.g. human geneticist, biologist, and physician) but were equally trained for the guidance of RD patients through the health care system. Participants were recruited by the Centre of Quality and Management in Health Care embedded in the State Medical Chamber of Lower Saxony in Hannover. All participants were recruited within the geographic region of Lower Saxony as this is regarded as representative of all areas of Germany. Only RD clinical guides were recruited all over Germany as they occur less frequently. The following selection criteria were employed: regional aspects were considered, differentiating professionals working in rural, urban, or metropolitan areas. Resident physicians were differentiated by whether their work was conducted in either single or joint practice. Regarding clinical doctors, the level of health care provision was considered, e.g. basic, regular, specialist, and maximum medical care. Finally, the hierarchy level of participants was considered, distinguishing between chief, senior, and assistant physicians.

### Ethical considerations

A positive ethics committee vote was obtained for the interview study from the ethics committee of the Albert-Ludwigs-University Freiburg (number 53/14). Informed consent was obtained in writing from all participants.

### Data generation and analysis

Semi-structured interviews were chosen as participants needed to be directed to the subject of interest. In some interviews, a narrative structure would have led to the extensive presentation of a single health issue, which was the focus of the person interviewed, not reflecting on other subjects, which were still important, though less so. Patient and relative interview flow was initiated by asking for experiences with diagnosis and treatment and important steps of their professional careers as well as experiences with RD patients on the side of HCPs. Then, interviewees were asked whether they saw a need for such a service. “How do you feel about the option to attain information by telephone?” If they were in favour of an RD helpline, they were encouraged to describe their mental picture of the helpline with particular reference to specific characteristics. A semi-structured interview guide was piloted during two interviews and afterwards adapted per interviewee needs. The HCP interview guide was developed in accordance with the structure of the guide for the people affected. However, some changes were necessary due to the different perspectives of HCPs on the topic. To ensure standardization, both interview guides were mutually discussed.

All interviews were recorded and later transcribed using the F4 transcription software. A standardized transcription guide was drafted for all interviews by three different interviewers. Transcripts were evaluated using MAXQDA, a programme for qualitative and mixed-methods data analysis. A structured content analysis was conducted following the guidelines provided by Mayring [72]. First, each interviewer formed categories inductively for three different interviews. Then, the chosen interviews were coded collectively to ensure inter-subjective or inter-rater reliability. Differences were addressed in the guide, clarifying a uniform coding strategy. Afterwards, attributes were extracted inductively by a single analyst to minimize interpretation bias. Finally, results were discussed within the whole research group, and results from patients and families were triangulated with those of HCPs. All quotations were translated by an external translation service, approved by a native speaker, and then included in the paper.

To ensure the quality of evaluation, the quality criteria of Mayring [73] were complied with.

## Results

Interviews were conducted until a high degree of saturation was achieved. No additional knowledge on RD information provision could be generated from further interviewing.

Following patients’ reports on predominant complaints of their complex diseases, all RD-affected interview partners could be categorised within one of the predefined disease groups. Patients with diseases of the digestive tract (*n* = 2), cystic fibrosis and lung diseases (*n* = 4), genetic diseases of the eye (n = 4), and disorders of the connective tissue (*n* = 5) were difficult to represent in the sample because of limited availability and polysystemic patterns. Therefore, the following patients could be included: genetic skin diseases (*n* = 10), skeletal dysplasia (*n* = 7), neuromuscular diseases (*n* = 9), genetic eye diseases (n = 4), connective tissue diseases (n = 5), genetic kidney diseases (*n* = 6), cystic fibrosis and pulmonary diseases (n = 7), congenital blood formation disorders (n = 4), immunodeficiency (n = 7), congenital metabolic disorder (n = 7) and genetic diseases of the digestive tract (n = 2). Participants could indicate disease severity on a three-item scale. Table 2 shows a summary of socio-demographic variables for patient and relative interviews.

**Table 2:** Socio-demographic variables, patients, and relatives

Sample characteristics		
Parameters	Patients and relatives (*n* = 68)	Physicians (*n* = 39)
Sex		
Male	23	23
Female	45	16
Age		
Average	51	46
Maximum/Minimum	85/18	
Educational qualification		
Abitur/A-levels	13	
Secondary education	19	
Technical collage/University	19	39
Advanced technical college degree	12	
Secondary modern school qualification	5	
Age at diagnosis		
Average	34	
Maximum/Minimum	74/0	
Disease severity		
No specification	4	
Low	8	
Medium	28	
Severe	28	
Profession		
Employed	31	39
Housewife/Houseman	2	
Unemployable/Special circumstance	15	
Student/Scholar	2	
Pensioner	18	

The table also shows socio-demographic characteristics of HCPs. One hundred and forty-one HCPs were invited to participate in the interview study. Of these, 39 candidates took part. Ensuring the diversity of participants, special regard was given to selection criteria concerning the structure of health care provision. Nine GPs, nine physicians, nine clinic doctors, six clinical guides, and six MTPs were included.

### Advocating for a rare diseases helpline

Of 55 RD patients, 41 advocated for the implementation of a helpline about RD. A smaller proportion (*n* = 8) objected to the service or described it as unnecessary as the telephone based information was already available online or through a physician. On the other hand, one interviewee interpreted the helpline as a tool for psychological counselling and neglected helplines in general for this reason. The remaining six participants did not offer a distinct answer. Regarding the interviews with relatives, 11 participants did endorse implementation. Two interviews could not be interpreted clearly as statements were given that were neither obviously for nor against an RD helpline. Therefore, a need for an RD helpline can be verified for some RD patients and relatives.

Of 39 HCPs, 18 endorsed the implementation of an RD helpline. Only four objected to the service or described it as unnecessary. An RD helpline was regarded as unnecessary whenever a suitable colleague was available. Physicians preferred a personal contact, they were already familiar with. A total of 17 participants offered an ambiguous or no answer. As physicians (*n* = 27) made up the largest portion of HCPs, these were evaluated separately as well. A total of 14 reported their endorsement of such a service, four claimed it to be unnecessary, and nine did not answer the question in a manner that could be definitively coded. Consequently, these findings verify for some HCPs a need for an RD helpline.

### Expectations towards a rare diseases helpline

A detailed summary of the content analysis of patient and relative interviews brought forth the following necessary characteristics of a helpline. Quotations are labelled by interview code, age, and gender. The interview code consists of a letter and interview number. The following abbreviations were used: ‘A’ standing for relative or affiliated, ‘P’ for patient, ‘GP’ for general practitioners, ‘S’ for specialists, ‘MTP’ for medical technical practitioners, and ‘G’ for guides.

### Staffing with professionals

Interviews demonstrated that patients and relatives perceived an RD helpline as helpful when their questions were answered by professionals. Ten of the participants explicitly highlighted this fact (*n* = 10). References to other access points, regardless of their profession, were experienced as rather unsatisfying.

*‘Currently it’s like this / our people are annoyed about it – they call up the branch office of LOCATION and have to ask their questions, then they call up LOCATION in order to receive an answer, and then they have to call back the people who are involved; I can’t conduct a conversation about my problems like this. I can’t solve a problem with a question – that usually functions in the most…./ or somehow, we’ll ask questions on Radio Eriwan, where the answers only consist of yes, no and occasionally perhaps.’ (P11/53/f).*

Similar to what was observed in the patient and relative interviews, HCP participants generally asked for a professional contact at the other end of the phone (*n* = 11). In most cases, a physician was named. One participant indicated that a hotline should not be staffed with a data management employee, nurse, or secretary even though they can sometimes be of much help.

*‘If one of them is clever and can give me tips afterwards, then I thank God for it and …/ but this should always come from doctors [I1: OK], not a nurse or a secretary*.” (GP02/37/m).

It was reasoned that only trained physicians could provide precise medical information. Therefore, an employee with substantive clinical experience was demanded. (S04/35/m) Expert knowledge of the person in charge was also highlighted. In the case of physicians, this meant extended training for one specialty. Nevertheless, biologists and laboratory experts were also mentioned in isolated cases. (GP05/61/m) Participants were also conscious of the difficulty of realizing this demand and therefore emphasized that an expert for each relevant medical field could not be demanded for an RD helpline. (S01/39/m).

### Personal contact

Another highlighted aspect was the importance of personal contact in addition to other rather impersonal information research systems (*n* = 10). Specifically, a single point of contact was demanded. It was reasoned that this kind of contact could accelerate and ease information search. Consequently, it was seen as helpful with regard to orientation in the health care system.

*‘Then they’ll surely sit down and study Internet sites and the brochures and information materials that are given out, but a human contact partner can sometimes expedite and simplify this search for information. Simply because one doesn’t just enter questions onto a screen by himself, but rather because he initiates communication with someone. If this office, the information office, was really staffed with competent personnel and not just some students who are completing their internship and don’t really know what it’s all about, then it would be a good idea, but would then also mean that money would need to be exchanged.’ (P37/46/m).*

On the other hand, psychosocial advantages were emphasised.

*‘Therefore, we have always sought out personal contact during the search and made use of it, simply because contact with a human being is much more pleasant and one can exchange information more effectively than when one simply calls up inflexible information from the Internet and then has to determine what is really applicable and what is not.’ (A02/48/m).*

The importance of personal contact (*n* = 7) was also identified as a category during HCP interviews. As expected, the focus was laid on the exchange of medically relevant information. For example, psychosocial issues were not named as a reason for the demand for direct communication. Instead, personal contacts were preferred as patients could be quickly introduced, and immediate feedback could be generated.

*‘Where one can also have a telephone conversation, which, in any case, is better than sending emails back and forth, since one can then react directly, briefly introduce the patient with his symptoms and perhaps even give the patient an appointment promptly, so that he can be examined in detail.’ (GP01/39/m).*

Frequently, an immediate contact and information receipt was required. (MTP02/35/f, MTP04/25/m, S04/35/m, GP01/54/f, GP03/48/f, C06/47/f) Furthermore, it was outlined that some medical issues cannot be described using predesigned web search masks given by Internet providers. Information can be searched only if previously made searchable. Fine nuances between blank facts cannot be depicted.

*‘So, to make a comparison once again; if I now say, as already mentioned in the example, I enter three things/ it’s different to saying to a colleague: “Man, I have the feeling that he’s really sick. And then it hurts somewhere on the left, sometimes more, sometimes less and so on”. It doesn’t make sense to enter this into a screen. [I1: Hm] And that’s really important.’ (GP02/37/m).*

### Availability

Participants (*n* = 6) expressed a wish for extended opening hours.

*‘And then, okay, if I have the office, let’s look at the ACHSE as an example. Then that’s also / and it’s rather stupid, at the one, they only work a half-day and it’s always … / so you always end up calling outside of business hours.’ (P06/85/f).*

Interviewees hope to avoid waiting periods and to receive contemporary answers. Waiting lines raise dissatisfaction and impatience (P17/47/f) similar to answering machines or automatic answers. (P51/62/f) It was reported that there should be at least enough human resource capacities to ensure a return call within an appropriate timeframe. (P14/57/f, P50/51/f).

HCPs referred to availability during four interviews (n = 6). HCPs did not highlight an uninterrupted 24/7 availability as important. In acute and/or life-threatening situations, an RD helpline would not be the first choice. In such a situation, an emergency call asking, for example, for a poison centre would be preferred. One GP mentioned that availability during regular office hours would be absolutely satisfying. Following the results of interviews with patients and relatives, it was also indicated that immediate availability is necessary, especially avoiding waiting lines.

*‘Personally, I find telephone conversations better, [I1: Hm] But I know how awful it is to be put on hold. [I1: Yes] [I2: Hm] Then one calls from here [I1: Yes] and tries to get connected. I know, I’ve had REALLY bad experiences there. If I want to reach anyone and I say to someone: I have five women here [I1: Hm] and then you get someone simply hangs on stubbornly. It can sometimes take HOURS. [I2: Hm, simply lay the receiver to the side] exactly! Lay the receiver to the side and wait until the call back comes through. That’s useless. [I2: Hm] I can’t afford to waste time like that here.’ (S04/35/m).*

This demand is in line with the demand for fast and immediate access to information. On the other hand, a dial-back system, collecting calls and answering them afterwards at a particular date, was also suggested by one participant (S13/50/m). Remarkably, this would contradict the demand for a fast access to information previously mentioned during interviews with patients, relatives, and HCPs.

### Low technical barriers

The telephone is also mentioned as an alternative to web access (*n* = 6) that is also suitable for the elderly and information seekers with no affinity for or no available Internet access. Additionally, one interviewee noticed that some people with RDs are limited in their mobility through their disease. Consequently, these people are unable to reach personal contact partners such as physicians and other therapeutic personal.

*‘However, the problem is often those people who can’t do it. We have a contact partner per telephone for those who are not mobile / great restrictions for the illness [AM]. Another example is the case of the DM 1 advanced stage, where the people are often no longer able to go places by themselves / they need so much strength and energy in order to cope with the few daily tasks, then they have something for it / but good, one always wants everything in any case.’ (P11/53/f).*

Asking HCPs for their opinion on the telephone as an alternative to the Internet as an information medium, results were heterogeneous. While younger HCPs preferred the Internet over a telephone and did not assign an important role to it, HCPs of higher age were rather indifferent or clearly preferred the telephone:

*‘Personally, I’m a big fan! [I1: Yes] So, the telephone— I would always give the telephone preference [LAUGHING], over some impersonal Internet site. But I think that’s also really “old-fashioned”’ (C07/42/f).*

HCPs even align with the need for a low technical barrier for certain patient sub-groups such as the elderly.

*‘Yes, I believe that exactly those people who, let’s say don’t have Internet access or who lack the knowledge, we’re talking about the older members of the public/. […]’ (C03/46/m).*

### Topics of counselling

Further, patients and relatives described possible topics that were expected to be discussed on the phone. Psychosocial and medical aspects were predominately named. Interviewees described the following medical contents: They hoped for an explanation of their disease pattern and of symptoms at hand.

*‘[…] first of all, the symptoms of the clinical picture, of course, and how the people affected deal with them. And then, of course, also self-help groups.’ (P52/39/f).*

In particular, participants demanded answers not only of general questions but also of questions concerning specific sections of the disease as well as information on the genetic background. (P12/58/f, P51/62/f) Concerning disease development information, possible methods to stop or lessen the burden of disease were reported to be most relevant. (P53/51/m) This was found in combination with the demand for information concerning the application of medication dosages or therapy and behaviour in the case of emergency. (P50/51/f, P54/40/m) Aside from these, patients also wished to be informed about the status of research. (P07/70/m).

In addition, persons concerned also brought forward psychosocial aspects. (A12/32/f, P47/59/m) Patients and relatives reported that they do see a need for the resolution of general problems arising from disability as well as specific disease problems. (P25/58/f, A05/60/f) Furthermore, it was perceived as helpful to talk about diseases, learn how other patients handle their disease, and learn whether self-help groups already exist. (P52/39/f) The importance of practical information on everyday life was highlighted again at this point. (A05/60/f) Just one person explicitly negated such an offer, claiming to be in no need of a helpline where one can have a good cry. (P04/39/m) On the other hand, a contact person was seen as an opportunity to counterbalance the desperation of one’s own situation with the prospect of being counselled and reserved when necessary. (P23/48/f) One interviewee noted that other sites did not take one seriously and hoped for an improvement. (A06/50/m) Similarly, when disorientated, a contact person was sought to aid with calming down, helping with the search, and coming up with concrete help.

*‘Yes, that one has a competent person on the other end of the line, so that one, for example, if he is doing badly or if he has any problems, that he receives the help he needs. In other words, that there is someone available for the moment. He doesn’t have to bring everything back into tip-top shape immediately. Just perhaps someone who is there to say: “Yes”, and “try to stay calm” for now, or, “I’ll help you, I will sort it out, I’ll do it” / “I’ll check up on it” and so forth, so that one isn’t simply/ yes, that one isn’t turned away, but rather… / or be subjected to long waiting times.’ (P21/53/f).*

When discussing topics of counselling, some HCPs specifically mentioned the need for endorsement concerning medical issues (*n* = 7). In particular, medical cases were reported as needing to be discussed via telephone, describing symptoms and patient histories. Three HCPs specified this demand, highlighting the need for differential diagnostics or a demand for assistance with the differential diagnostic process of elimination. (GP03/46/f, MTP04/25/m, S04/35/m).

*‘I would also think that this could be useful for rare diseases, so that one could simply receive a differential diagnosis, a second opinion. So, I’ll tell you what the symptoms are and you can tell me what it could be.’ (MTP04/25/m).*

Participants also demanded information on self-help groups. (GP05/61/m).

### Guidance

Those polled also talked about the necessary functions of a helpline. Often, aiding orientation within an information overflow or during information undersupply seemed to be necessary. Additionally, the sample demanded a guide to lead the way through information chaos. (P09/47/m) Beyond that, advice for further research was seen as beneficial. (P52/39/f, P29/44/f, P38/60/f) Even a general reference suggesting that such information exists was perceived as helpful. (P13/54/f) Therefore, it is not surprising that the scope of available information was most commonly underestimated.

*‘Although sometimes one naturally also …/ one thinks he is well informed, and he has no idea that there is actually still much, much more information available or that a variety of other opportunities exist for him.’ (P32/40/f).*

This category did not occur during HCP interviews.

### Referral

Another function that was additionally demanded was referral. For example, information about care facilities and physicians was cited (A06/50/m, P10/50/f), indicating that this is of special importance at the beginning of a disease. (P47/59/m) Nevertheless, it was also highlighted that this was not the only task.

*‘A12: Yes, I find it good (info hotline). But, in my opinion, as I have just indicated, that would need to be a little larger. That psychosocial counselling services are referred to.*

*Interviewer: Yes, OK.*

*A12: and that one does more than to just say, “Yes, there’s the doctor.”’ (A12/32/f).*

HCPs also mentioned the need for referral in addition to medical counselling (*n* = 3). At this point, HCPs reported that they realize that it is impossible to make their wish for immediate specialist knowledge for each medical field come true. On the contrary, they realize the impracticability of this demand.

*‘That makes sense, yes. That makes sense. Well, I wouldn’t expect to be able to call the medical association, for example, and say that I have someone on the phone who is experienced in this area. [I1: Hm] You can’t expect that. But if you can call and say: ‘Do you have a contact that is particularly responsible for such and such a disease pattern’? That makes sense.’ (S01/39/m).*

*‘Let me say, in order to be in a position to address his request, and I believe that this telephone opportunity is really good here, since it gives us the opportunity to shift and sort a little and [I1: okay] to say who belongs here and who doesn’t.’ (G07/31/f).*

In this regard, HCPs emphasize that the number of referrals can and must be minimized to shorten odysseys through health care systems. (GP03/48/f).

## Discussion

Patient and relative interviews showed that helplines are predominantly necessary due to the possibility of personal contact and low technical barriers. RD patients and relatives wish for a helpline run by professionals with extended availability. An RD helpline should offer information on medical and psychosocial issues. In addition, participants hope for guidance through information chaos as well as referral where needed.

In general, the need for an RD helpline from the perspective of HCPs was confirmed with some minor differences, even though no statements to the extent of the demand can be made to a comparably high percentage of unspecific answers. An RD helpline should be staffed with professionals. However, a medical professional was specifically demanded. Criteria for staffing should be broad knowledge of RD, a multidisciplinary orientation, and knowledge of differential diagnostic procedures. Personal contact was preferred as details of medical cases could be described, even if not put in words easily. HCPs also asked for additional referrals to other experts. Good availability was specified as reachability during office hours suggesting that a request surplus could be managed through a call-back system. As this proposal is not in line with patient and relative interviews, it is not considered for the final concept. Medical professionals recognized a low technical barrier as an important issue for themselves and people affected.

Many studies report the staffing of helplines with nurses [74]. In this study, patients, family members, and physicians particularly demanded the employment of professionals with a special emphasis on physicians. McKenzie, [75] for example, reported the successful commitment of GPs for the coverage of after-opening hours. In this regard, the broad knowledge of GPs seems to be a suitable qualification for the management of an RD helpline potentially incorporating the need to familiarize with various diseases across different medical disciplines. Besides, GPs are familiar with transferring patients and communicating with various medical professionals. GPs also add their expertise when it comes to long-term differential diagnostics. On the other hand, training can add to the necessary qualification spectrum especially when it comes to very rare diseases or psychosocial needs and seem to be quite suitable for the management of helplines even though a re-alignment of practice is necessary due to the interaction via telephone [75]. Advice has been obtained by the German Cancer Information Center which offers a German Cancer Helpline. In this context, psychologist or social workers are often added to the Team rendering advice to other counsellors [76–78].

The importance of personal contact was highlighted during the interviews. Even though interviews raised ease of information search as an argument for building a helpline, the psychological value of personal contact also needs to be stressed. Anderson [79] raised a high negative impact even on family members of children suffering from RD and demanded more psychological support. Helplines salvage the potential of reducing this distress [67] and therefore offer high psychological value.

Availability is a very subjective topic. As RDs mostly show a chronic pattern, emergency situations will not arise that often. Emergency calls can be addressed to emergency helplines. Nevertheless, the demand for extended opening hours remains unclear in its specification and requires further discussion.

Even though we live in a technically advanced digital age, there are still some EU households with no Internet connection (32%). On the other hand, 98% have telephone access, either through a fixed or mobile device [80]. Adding to this, telephone services are always available in local languages while many websites on RDs are only available in English language, adding a language barrier to the technical one [67]. Nevertheless, it must be outlined that these barriers will be further reduced as technologic advancement progresses. Besides, there are already some translation programmes available online, which will most certainly be further developed.

Friedmann et al. [81] confirm the necessity of a multidisciplinary team for the coverage of inquiries of callers of a HCP helpline and therefore underpin the broadness of questions.

Originally, primary care physicians took on the role of guides when communicating and assessing health information. Therefore, it is not surprising that this category did not emerge within HCP interviews as they identify with this role. Nevertheless, Coumou and Meijman [82] state that GPs do have approximately 400 indications at hand. It is very likely that common indications are kept in mind rather than RDs, which are very unlikely to appear in their practice. These findings underpin the role of guides, which already exists as part of many RD centres and whose expertise is demanded in this case.

Tariq et al. [83] confirm the importance of referrals carried out by helplines. It quantifiably contributes to the effectiveness of health care systems. For example, an after-hours service helpline prevented 1363 people from unnecessarily attending an emergency department. Further, 228 individuals underestimating their conditions could be referred to an adequate health service provider.

### Study significance

We suggest that our study has significance for the establishment of nationwide and centralized RD helplines worldwide due to shared problems such as long delays in diagnosis and dense RD health care infrastructure. In addition, the study broadens the perspective on RD telephone services rendered within the literature thus far by including potential users who have not yet called a helpline but would if services were adapted. In contrast, previous studies interviewed callers of existing helplines, focusing on affected people who were already interested in the service of the helpline [61, 64]. This new perspective offers a way to improve RD counselling, making it more attractive to the potential user pool and, therefore, extending its benefits to all those affected within society. Besides, many studies dealing with the question of health information provision do not include the possibility of different information access points. However, existing studies—for example, Mooney et al. [84]—found that patients suffering from anti-neutrophil cytoplasmic antibodies tended to reject detailed information on their disease and disease management when given the diagnosis through a physician as it was a lot to take in. At a later stage, truthful information was difficult to access, substantiating the benefit of a telephone service. Other studies analysing patients and families dealing with late stage cancer underpin the assertion that trained physicians may not communicate effectively due to missing knowledge of information needs of this patient group, [85] indicating the need for specialized and broadly available service providers.

Most heatedly discussed was the implementation of a central RD helpline considering all 5000 or 8000 very heterogeneous diseases. Implementing this kind of service necessitates an extensive financial budget. The estimation of necessary financial resources proves to be quite difficult as many assumptions and projections are necessary. A high-budget case with 60,589 estimated contacts per year necessitates an annual budget of 2.59 € million with 35 full-time employees (FTE).^1^ As full case coverage requires extensive budgeting, the calculation is rather an indicator for what is already done for other diseases and could be done in the field of RD. However, a competent counselling service can be offered. In this case, the overall estimated need for RD information need cannot be covered. Costs for a base case scenario mount up to approximately 300.000 € (4 FTE) annually.^2^ An evaluation of European telephone services by Houÿez et al. [53] shows similar results. According to the report, RD helplines should be staffed with a minimum of 1.5 (FTE), leading to annual costs of 150,000 to 300,000 €. Therefore, it can be suggested that, starting from this level, a stepwise implementation of the ideal scenario should be pursued.

As a solution for the shortage of monetary funds, a central telephone service offering referrals is often suggested. Such a service could bundle the heterogeneous landscape of existing RD telephone-based or disease-related information services in a similar manner to how ZIPSE is bundling web information. However, the implementation of such a service would contradict the results of the study as patients, relatives, and HCPs ask for direct contact with professionals. Therefore, a telephone service bundling all RD helplines and giving references cannot be suggested. Nevertheless, it can be suggested that existing RD-related helplines may be shaped following the results of this interview study. For example, the service of the Alliance of Chronic Rare Diseases (Allianz chronisch seltener Erkrankungen, ACHSE) can be further extended. Services of RD guides located at specialized centres for RDs can be adapted, bearing thoroughly in mind the wish of patients and relatives to not only be forwarded from one contact point to another.

### Assumptions and limitations

This study was designed qualitatively to capture information needs, which could be served using a telephone service without guiding answers beforehand. Instead, participants were encouraged to give their own ideas on an RD helpline, assuming these to be of most relevance. Therefore, a limited number of patients, relatives, and HCPs could be interviewed. The qualitative design contributes to theory generation. The quantitative structure of interview results has been included to increase the transparency of result communication. To make projections and/or quantifiable statements, results need to be verified through a quantitative study.

Only 39 HCPs participated in the study from 141 invited. Studies show that physicians are more likely to respond when a small financial incentive is given. During this study, no financial resources were available for this purpose [86].

Many female individuals were interested in participating in the study. That is why the sample is biased towards women. Even though this should be kept in mind, studies show that health information providers are more often used by women as they are more likely to search for health information in general. Some providers report up to 97.5% female users [87].

The study was conducted against the backdrop of the German NAMSE process asking for the design of a national RD helpline. In order to minimized the bias towards favouring the establishment of a RD helpline interviewers first openly asked how participants feel about helplines to avoid putting neither negative nor positive words into the mouth of participants as suggested by Mayring 2002 [72]. Therefore, participants were not influenced towards a specific outcome.

Additionally, patient and relative interviews were conducted by three different interviewers. HCP interviews were held by two different interviewers partially conducting interviews together. Even though interview structure was discussed beforehand and interview guides were established and adapted after piloting, individual interview styles need to be recognized as an influential factor.

Interviews were not able to capture juridical topics during the questioning concerning the helpline. Therefore, it is obviously necessary to analyse why respondents did not include juridical or access to treatment matters even though experiences of other helplines show that people affected do not solely search for this via other media [63]. ACHSE user statistics (2011–2013; unpublished, based on private email communication) indicate that problems with cost takeover and other social legal problems are topics of counselling. Independent Patient Consultancy (Unabhängige Patientenberatung Deutschland, UPD) reports proportions of 66% and 67% [78, 79] medical-juridical questions within their annual patient monitor, pointing to the most likely reason for not mentioning juridical issues during the interviews. Obviously, they are closely linked to medical questions and not visible at first glance using structured content analysis.

## Conclusions

Even though new technologies enable patients, relatives, and HCPs to access information rapidly, this study shows that there is still a point in making information accessible the ‘old-fashioned way’ via telephone. The telephone offers the unique chance to make professional insights directly available for all stakeholders, including exchanging medical and psychological issues. However, putting all desired aspects simultaneously into practice in an ad hoc implementation process with a central RD helpline offering information for all patients, relatives, and HCPs potentially calling the helpline would necessitate a huge financial budget. Therefore, a stepwise implementation is suggested. As a first step, it is suggested to improve major existing helplines to meet the identified needs. Afterwards, service availability can be extended. In the long run, existing services should be evaluated with regard to the fulfilment of these factors. The expertise from institutions as centres for RDs should be further included, bearing in mind the wish of patients and relatives to not be pushed from one information access point to another.

## Abbreviations

A: Affiliated or relative
ACHSE: Allianz chronisch seltener Erkrankungen, Alliance of Chronic Rare Diseases
C: Clinician
ENRDHL: European Network of Rare Disease Help Lines
FTE: Full-time employees
G: Guide
GMS: German Medical Science
GP: General Practitioner
HCP: Health Care Professionals
KID: Krebs Informations Dienst, German Cancer Information Service
MTP: Medical Therapeutic Practitioner
NAMSE: Nationales Aktionsbündnis für Menschen mit Seltenen Erkrankungen, National Action League for People with Rare Diseases
P: Patient
RAPSODY: European Rare Disease Solidarity Project
RD: Rare Diseases
S: Specialist
UPD: Unabhängige Patientenberatung Deutschland, Independent Patient Consultancy

## Footnotes

1 In an ideal scenario, all identified relevant aspects should be put into practice. This first scenario demonstrates how many monetary resources could be put into a telephone-based information service when aiming for a service quality similar to that of the UPD and the KID. User statistics of the KID show that only 1.51% of the patient pool uses the information service, resulting in 23,024 contacts per year [78]. Extrapolating from this number, an RD helpline would need to expect 60,589 requests per year, making 35 employees necessary to answer all requests [76–78]. Average human resource costs of an interdisciplinary team are estimated to be 59,006.61 € per employee and year. Costs were calculated including monetary funds for rent, staffing, office equipment, publicity, and employee development. Calculating overall material costs makes an estimation of material cost per person possible, approximately 14,911.20 € in the initial year and 12,907.83 € for the following years. The calculation is based on German average wages of a mixed team consisting of physicians, social workers, and lawyers. Accordingly, an RD helpline makes funding of 2.59 € million in the first year necessary.

2 Because of limited budgets, a low-budget case has been discussed as a second scenario. Following interview results, the most important aspect, ‘quality of counselling by experts’, shall be obtained. Therefore, a multidisciplinary team shall be preserved, aiming for a minimum staffing by one physician, one social worker, one lawyer, and one temporary employee. Hence, a quality counselling service can indeed be offered, but, comparing both cases, the counselling team in the latter case is significantly shorthanded and therefore unable to cover all potential requests. In a base-case scenario, considering four full-time employees, overall material costs add up to 59,644.80 € for the initial year. For the following years, 51,631.30 € in costs are estimated. As previously mentioned, average human resource costs add up to a total of 236,026.44 €. Finally, the calculation results in overall costs of 295,671.24 € for the first year and 287,657.74 € for the following years.
